# Infection of human Nasal Epithelial Cells with SARS-CoV-2 and a 382-nt deletion isolate lacking ORF8 reveals similar viral kinetics and host transcriptional profiles

**DOI:** 10.1371/journal.ppat.1009130

**Published:** 2020-12-07

**Authors:** Akshamal M. Gamage, Kai Sen Tan, Wharton O. Y. Chan, Jing Liu, Chee Wah Tan, Yew Kwang Ong, Mark Thong, Anand K. Andiappan, Danielle E. Anderson, De Yun Wang, Lin-Fa Wang

**Affiliations:** 1 Programme in Emerging Infectious Diseases, Duke-NUS Medical School, Singapore; 2 Department of Otolaryngology, Infectious Diseases Translational Research Programme, Yong Loo Lin School of Medicine, National University Health System, National University of Singapore, Singapore; 3 Department of Otolaryngology, Head & Neck Surgery, National University Health System, National University Hospital, Singapore; 4 Singapore Immunology Network (SIgN), A*STAR, Singapore, Singapore; 5 Singhealth Duke-NUS Global Health Institute, Singapore; Johns Hopkins University Bloomberg School of Public Health, UNITED STATES

## Abstract

The novel coronavirus SARS-CoV-2 is the causative agent of Coronavirus Disease 2019 (COVID-19), a global healthcare and economic catastrophe. Understanding of the host immune response to SARS-CoV-2 is still in its infancy. A 382-nt deletion strain lacking ORF8 (Δ382 herein) was isolated in Singapore in March 2020. Infection with Δ382 was associated with less severe disease in patients, compared to infection with wild-type SARS-CoV-2. Here, we established Nasal Epithelial cells (NECs) differentiated from healthy nasal-tissue derived stem cells as a suitable model for the *ex-vivo* study of SARS-CoV-2 mediated pathogenesis. Infection of NECs with either SARS-CoV-2 or Δ382 resulted in virus particles released exclusively from the apical side, with similar replication kinetics. Screening of a panel of 49 cytokines for basolateral secretion from infected NECs identified CXCL10 as the only cytokine significantly induced upon infection, at comparable levels in both wild-type and Δ382 infected cells. Transcriptome analysis revealed the temporal up-regulation of distinct gene subsets during infection, with anti-viral signaling pathways only detected at late time-points (72 hours post-infection, hpi). This immune response to SARS-CoV-2 was significantly attenuated when compared to infection with an influenza strain, H3N2, which elicited an inflammatory response within 8 hpi, and a greater magnitude of anti-viral gene up-regulation at late time-points. Remarkably, Δ382 induced a host transcriptional response nearly identical to that of wild-type SARS-CoV-2 at every post-infection time-point examined. In accordance with previous results, Δ382 infected cells showed an absence of transcripts mapping to ORF8, and conserved expression of other SARS-CoV-2 genes. Our findings shed light on the airway epithelial response to SARS-CoV-2 infection, and demonstrate a non-essential role for ORF8 in modulating host gene expression and cytokine production from infected cells.

## Author summary

Airway epithelial cells are one of the earliest cell types infected in COVID-19 patients. We show that differentiated NECs are a suitable model for studying the dynamics of SARS-CoV-2 infection *in vitro*, and that the immune response from infected NECs is surprisingly limited. This limited early response to SARS-CoV-2 infection could impair viral clearance, and prolong the duration of infection. This further implies that infiltrating immune cells are the likely source of pro-inflammatory cytokines such as IL-6 and TNFα reported to be elevated in patient sera later during infection. CXCL10 production could represent a major therapeutic node for limiting immune cell infiltration and subsequent cytokine production. The similarities in host-response between SARS-CoV-2 and Δ382 infection *in-vitro* highlight the plasticity of CoV genomes, and implicate a post-translational (and not a transcriptional) role for ORF8 in modulating the host-response.

## Introduction

The novel coronavirus SARS-CoV-2 was first detected in China in late 2019, and has since spread rapidly across the globe [1,2]. The high transmissibility of this virus as well as the ability to cause serious disease has resulted in a heavy healthcare and economic burden. While generally manifesting as a mild upper respiratory tract infection, SARS-CoV-2 is able to cause severe lower airway disease including pneumonia and acute respiratory distress syndrome (ARDS) in a variable fraction of patients [3,4]. Understanding the host-protective mechanisms responsible for viral clearance, and the related identification of causative factors leading to the development of severe disease vs asymptomatic infection are presently the subject of intense research.

Airway epithelial cells, in particular those of the nasal epithelium, are postulated to be the first site for viral contact during the establishment of SARS-CoV-2 infection in a new host [5,6]. Besides providing a physical barrier to host infection, epithelial cells are proficient in mounting an immune response to infection. This includes the expression of antiviral factors upon pathogen detection by cellular pattern-recognition receptors, as well as the secretion of cytokines and chemokines which can recruit and activate both the innate and adaptive arms of the immune system [7]. As the early response from this initial site of infection can play a major role in determining the subsequent trajectory of disease development, there is an important need to establish model systems for studying the epithelial immune response to SARS-CoV-2.

SARS-CoV-2 is a *Betacoronavirus* that belongs to the *Coronaviridae* family, a group of single-stranded, positive-sense RNA viruses. Unlike other RNA viruses such as influenza, CoVs possess proofreading mechanisms, thus accumulating mutation at a slower rate [8,9]. Global sequencing efforts during the course of the COVID-19 pandemic have nevertheless identified viral variants and mutational hotspots in the SARS-CoV-2 genome [10–14]. Thus far, the most notable SARS-CoV-2 variant carries a D614G mutation in the spike protein and has become the most prevalent strain across geographical locations, indicating a likely fitness advantage [15]. Monitoring the occurrence of SARS-CoV-2 variants, and assessing their impact on disease outcomes and transmission are important for informing public health measures during the pandemic.

A 382 nucleotide deletion variant (Δ382) was recently isolated in Singapore [14], and also reported in surveillance studies in Taiwan [16]. Deletion of this genomic region results in a truncated ORF7a, and absence of ORF8 expression due to lack of most of the ORF8 gene and loss of the ORF8 transcription-regulatory sequence [14,17]. Infection with the Δ382 variant has been associated with milder infection [17]. Notably, none of the patients in which the Δ382 variant were detected required intensive care unit admission or invasive mechanical ventilation, compared to 16% and 11%, respectively, for the wild-type strain [17]. However, detailed comparative studies upon *in-vitro* infection with wild-type SARS-CoV-2 and Δ382 are still lacking. Here, we established human NECs (hNECs) as a platform to study the pathogenesis of SARS-CoV-2, and utilized this system to compare viral replication kinetics and host responses between wild-type SARS-CoV-2 and Δ382 infection *in-vitro*.

## Results

### ACE2 and TMPRSS2 are expressed on human upper airway tissue and differentiated NECs

**Fig 1:**
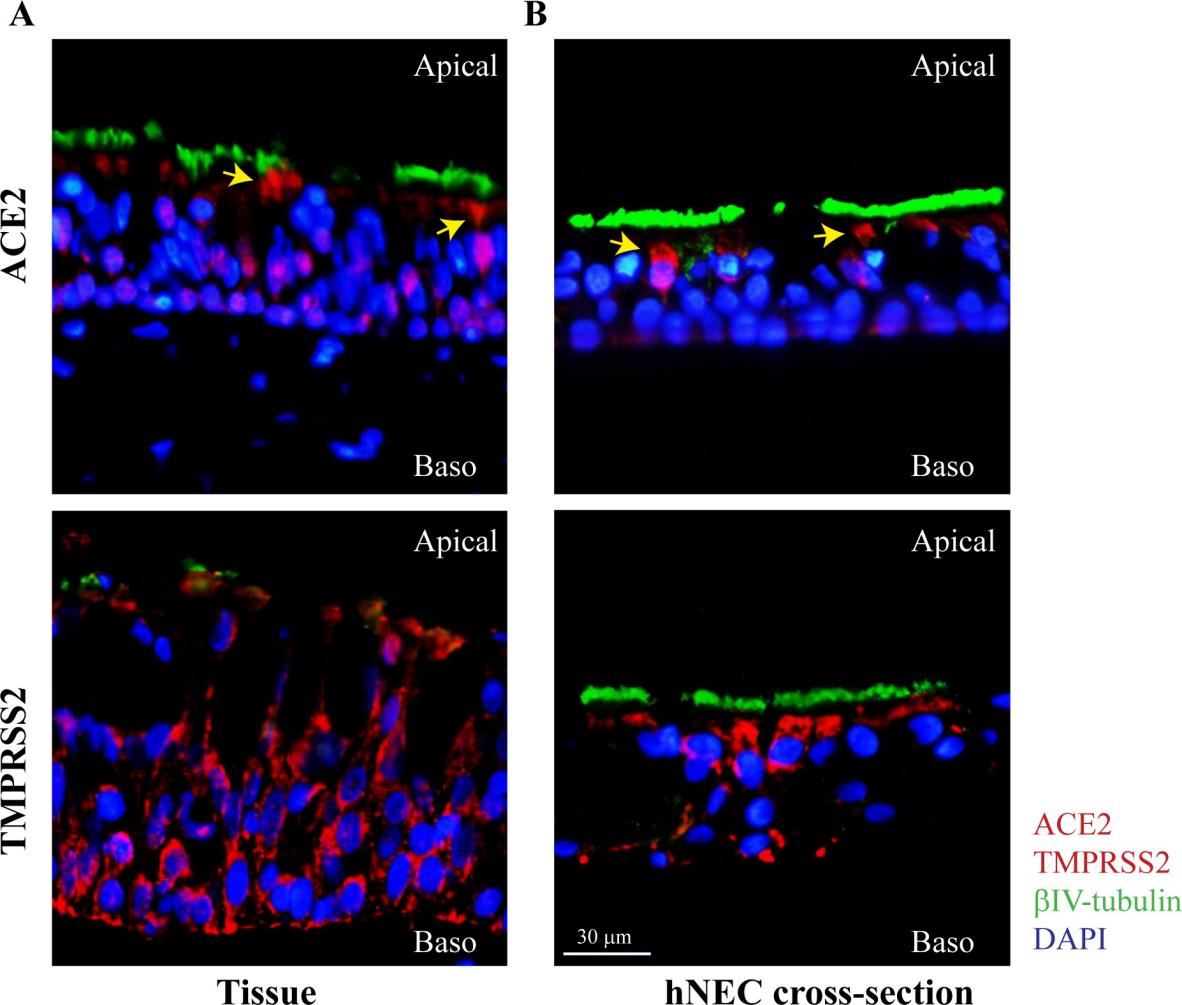
Expression of SARS-CoV-2 entry factors on primary nasal tissue and differentiated NECs. Immunofluorescence staining for expression of ACE2 and TMPRSS2 on (A) primary nasal tissue, and (B) cross-section of NECs. ACE2 or TMPRSS2 staining in the relevant panels are represented in red, βIV-tubulin in green, and nuclear staining with DAPI in blue. Representative images from at least two independent stains are shown, at a magnification of 400x.

Immunofluorescence staining of upper airway tissue sections confirmed the protein-level expression of SARS-CoV-2 entry factors ACE2 and TMPRSS2 (Fig 1A). ACE2 appeared more prominently on the apical side of the epithelium (Fig 1, indicated with yellow arrows), while TMPRSS2 was expressed uniformly throughout the upper airway epithelium (Fig 1A). Our data is in agreement with reports on the expression of SARS-CoV-2 entry factors in human upper airway tissue [5] and supports the high transmissibility of SARS-CoV-2. We next generated *in vitro* differentiated hNECs from stem-cells derived from donor biopsy samples, according to protocols established by our group previously [18]. Similarly to primary tissue, ACE2 and TMPRSS2 expression was detected on differentiated hNECs, with ACE2 distribution skewed towards the apical side (Fig 1B). Staining controls with the secondary antibody alone demonstrated the absence of non-specific staining (S1A and S1B Fig). To further validate the specificity of antibody staining, VeroE6 and HEK293T cells transfected with plasmids over-expressing human TMPRSS2 and ACE2, respectively, were stained for immunofluorescence assay (S1C and S1D Fig). TMPRSS2 and ACE2 staining were only observed within samples transfected with the relevant ORFs (S1C and S1D Fig). In conclusion, these data indicate that both primary nasal tissue and differentiated hNECs readily express entry factors required for SARS-CoV-2 infection.

### NECs are permissive to SARS-CoV-2 infection, with comparable virus release between SARS-CoV-2 and Δ382

**Fig 2:**
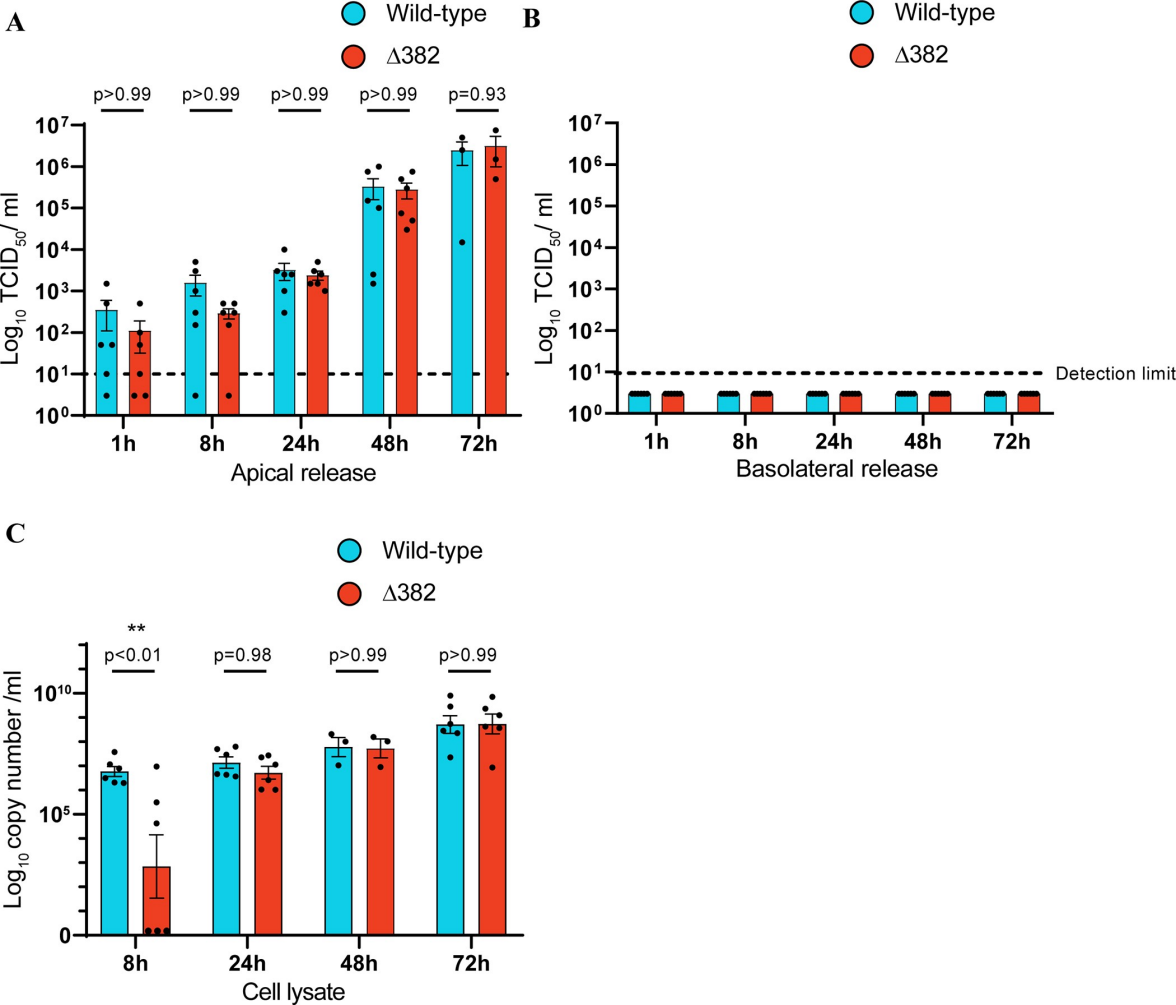
SARS-CoV-2 and Δ382 replication kinetics in NECs. Virus particles released from the (A) apical and (B) basolateral surface of infected NECs, and (C) virus copy numbers detected in cell lysates, of infected NECs at each of the indicated time-points (n = 3–6). Each dot represents a different human-donor derived NEC. Data represented as mean ± SEM. Adjusted p-values derived from two-way ANOVA test with Sidak correction are indicated above each comparison.

NECs were infected with SARS-CoV-2 and Δ382 at an MOI of 0.1. Apart from the deleted genomic region in Δ382, there are three amino acid changes between the two strains, in S, ORF3A and M. There are no differences in the RBD domain of spike protein, or in the furin-cleavage site (S1 Table). Infection resulted in the gradual release of virus from the apical surface, with no virus particles detected in the basolateral compartment up to 72 hours post-infection (hpi) (Fig 2A and 2B). No significant difference in virus titer from SARS-CoV-2 and Δ382 infected NECs was observed at any of the time-points examined. A cumulative increase in virus copy number was also observed in cell lysates, with similar kinetics for both strains except at 8 hpi, when Δ382 infected NECs had lower viral copy numbers (Fig 2C). Therefore, while Δ382 exhibits a minor lag in viral RNA during early replication, both wild-type and Δ382 were exclusively released from the apical surface of infected NECs, at comparable levels.

### Basolateral secretion of CXCL10 from NECs in response to infection

**Fig 3:**
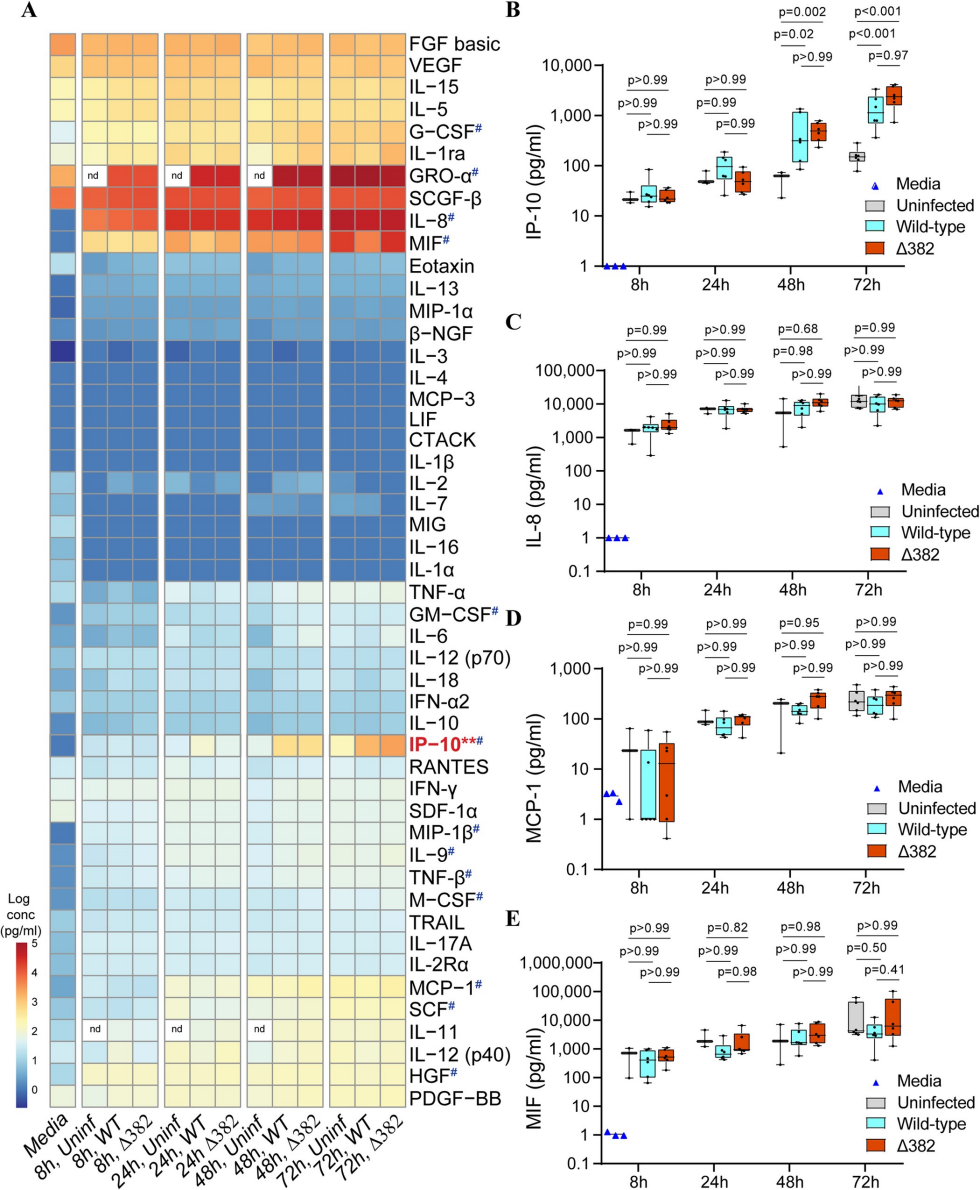
Cytokine secretion from NECs upon SARS-CoV-2 and Δ382 infection. (A) Heat-map of average cytokine concentrations (pg/ml, log) detected at the indicated time-points and infection conditions. All cytokine values are averages of 6 independent donor-derived NECs, except for GRO-α and IL-11, which represent average values from 3 independent donor-derived NECs. # indicates cytokine(s) significantly secreted by NECs in the uninfected state (adjusted p-value < 0.01, two-way ANOVA test with Tukey correction and > 10 fold increase in cytokine signal compared to media only control). ** indicates cytokine(s) concentration significantly increased upon infection, compared to uninfected samples (adjusted p-value < 0.01, two-way ANOVA test with Tukey correction), nd: not done. Box and whiskers plot of cytokine concentrations (pg/ml, log) for (B) IP-10 (CXCL10), (C) IL-8, (D) MCP-1 and (E) MIF, at each of the indicated time-points and infection conditions (n = 6). Each dot represents a different human-donor derived NEC. Adjusted p-values derived from two-way ANOVA test with Tukey correction are indicated above each comparison.

An excessive cytokine response is associated with the development of severe COVID-19 symptoms, and higher levels of several pro-inflammatory cytokines have been detected in patient sera. To understand the cytokine profile secreted from infected airway cells into the blood vessels and submucosal tissue of COVID-19 patients, we assayed the basolateral compartment from infected NECs for a panel of 49 cytokines. We observed that CXCL10 was the only cytokine induced upon SARS-CoV-2 infection, with significantly higher levels of cytokine observed from infected samples at later time-points, compared to uninfected controls (Fig 3A and 3B). Importantly, no difference in CXCL10 production was observed between SARS-CoV-2 and Δ382 at any of the time-points (Fig 3B). Several cytokines and chemokines, including IL-8, MCP-1, and MIF were observed to be basally secreted from NECs at significant concentrations, consistent with dynamic communication between airway epithelial tissue and the immune system even in the unperturbed state (Fig 3A and 3C–3E). Taken together, we demonstrate that CXCL10 is an important cytokine secreted from infected airway epithelial cells, and that these cells are unlikely to be the source of classic pro-inflammatory cytokines such as IL-6 and TNFα reported to be elevated in the systemic circulation of COVID-19 patients.

### Temporal regulation of distinct gene-sets after SARS-CoV-2 infection

**Fig 4:**
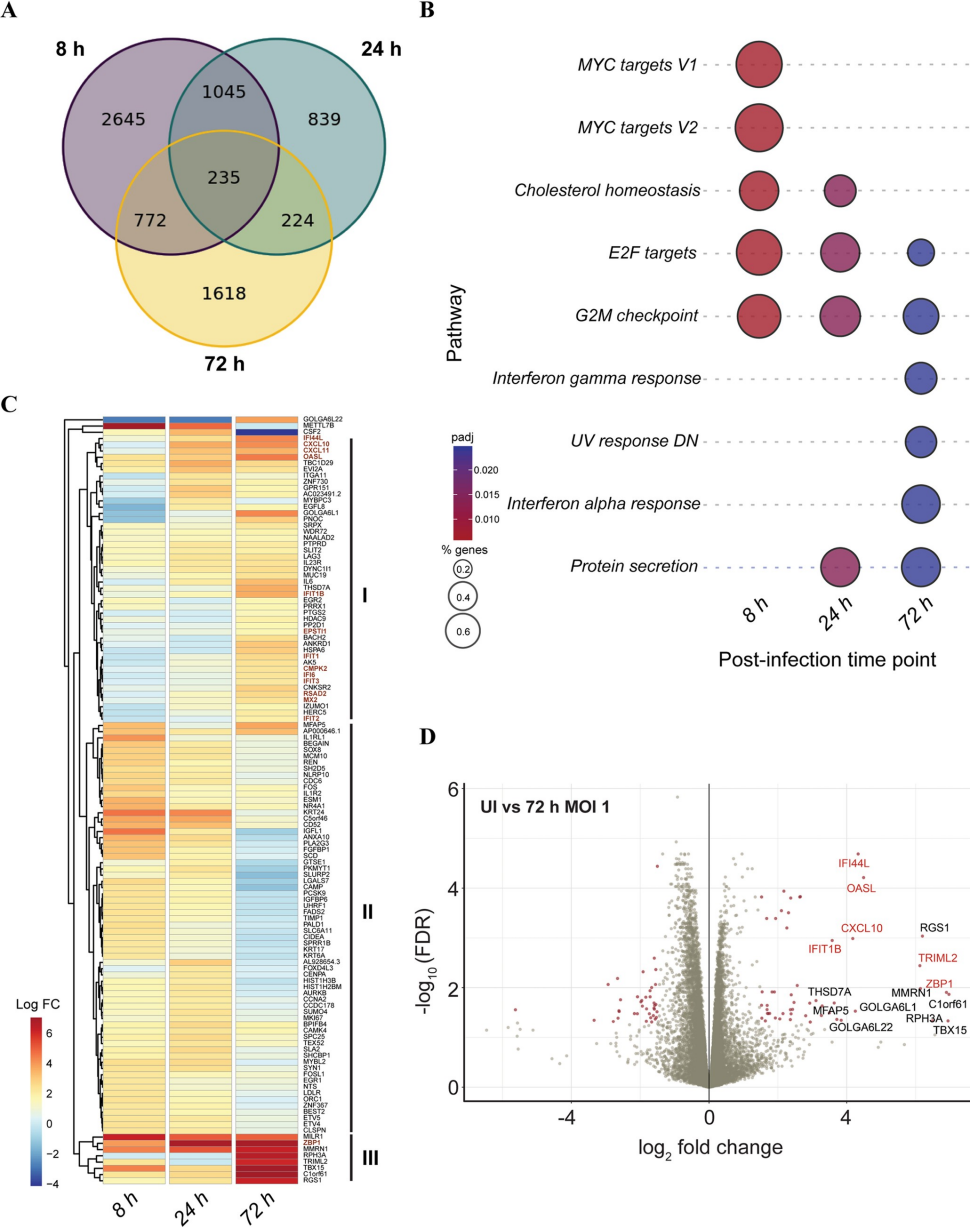
Transcriptional response to SARS-CoV-2 infection from NECs. (A) Venn diagram of shared or unique DEGs upon infection of NECs with wild-type SARS-CoV-2 at each of the indicated time-points. (B) Bubble-plot of top enriched Hallmark gene-sets upon infection of NECs with wild-type SARS-CoV-2. The color and size of each bubble is proportional to the adjusted p-value and the percentage of enriched genes from each gene-set, respectively. Only gene-sets with an adjusted p-value < 0.05 are shown (C) Heat-map of top 50 up-regulated DEGs observed at 8 h, 24h and 72 h after infection of NECs with wild-type SARS-CoV-2. Genes which correspond to Hallmark Interferon Alpha response, or Gene Ontology term Response to Type I Interferon are colored in red. (D) Volcano plot of genes at 72 h after infection of NECs with wild-type SARS-CoV-2. Significant DEGs with an adjusted p-value < 0.05 and log FC of at least 1.5 are indicated as maroon dots; all other DEGs are indicated as grey dots. The top 15 up-regulated genes by log FC are annotated, with interferon-stimulated genes annotated in red.

We next analyzed the transcriptional response to SARS-CoV-2 infection in NECs at 8, 24 and 72 hpi. Most differentially expressed genes (DEGs) during the early (8 hpi) and late (72 hpi) stages of infection were observed to be specific to those respective time-points (Fig 4A). This was confirmed by an examination of the top gene-pathways enriched at early and late time-points by gene-set enrichment analysis (Fig 4B). Genes corresponding to Myc targets, E2F targets, G2M checkpoint and cholesterol homeostasis were enriched during early infection. Conversely, genes associated with an interferon alpha and gamma response were only observed to be enriched at 72 hpi. Unsupervised clustering of the top up-regulated genes from each time-point demonstrated two distinct temporal patterns of gene regulation after SARS-CoV-2 infection (Fig 4C). Genes in Clade I and III showed a generally increased expression over time, while genes in Clade II decreased in expression over time (Fig 4C). Notably, genes linked to a Type I IFN response were specific to Clades I and III (in red). To explore variation in the immune response between donors, the fold change values for genes linked to Type I IFN response were re-calculated for each individual donor and plotted as a heat map (S2A Fig). While the overall trend of interferon-related gene induction upon infection was conserved, donor-specific differences in induction patterns were observed. In particular, viral output was observed to negatively correlate with robust interferon-related gene induction at early time-points (S2B and S2C Fig). Although we are unable to make any specific conclusions due to the limited sample size, a donor-derived NEC model for SARS-CoV-2 infection can have utility in further studies to interrogate patient-specific sources of variation that can impact both viral replication and the host immune response. Taken together, these data demonstrate that SARS-CoV-2 infection results in a dynamic epithelial cell transcriptional response, including the clear induction of antiviral genes associated with interferon signaling (Fig 4D), although only at late time-points post-infection.

### SARS-CoV-2 induces an attenuated inflammatory response upon NEC infection compared to Influenza A subtype H3N2

The host transcriptional response to SARS-CoV-2 was compared with that induced by another respiratory virus, Influenza A subtype H3N2. H3N2 is a common strain of seasonal influenza, and generally causes mild, uncomplicated upper respiratory tract disease, with complications occurring in a minority of the patients [19]. Influenza A and CoVs are single-stranded RNA viruses, and thus would share overlapping detection by host pattern-recognition receptors. We utilized a previously published dataset by our group, in which human NECs were infected with H3N2 at an MOI of 0.1 under similar conditions to SARS-CoV-2 infection in this study [20].

**Fig 5:**
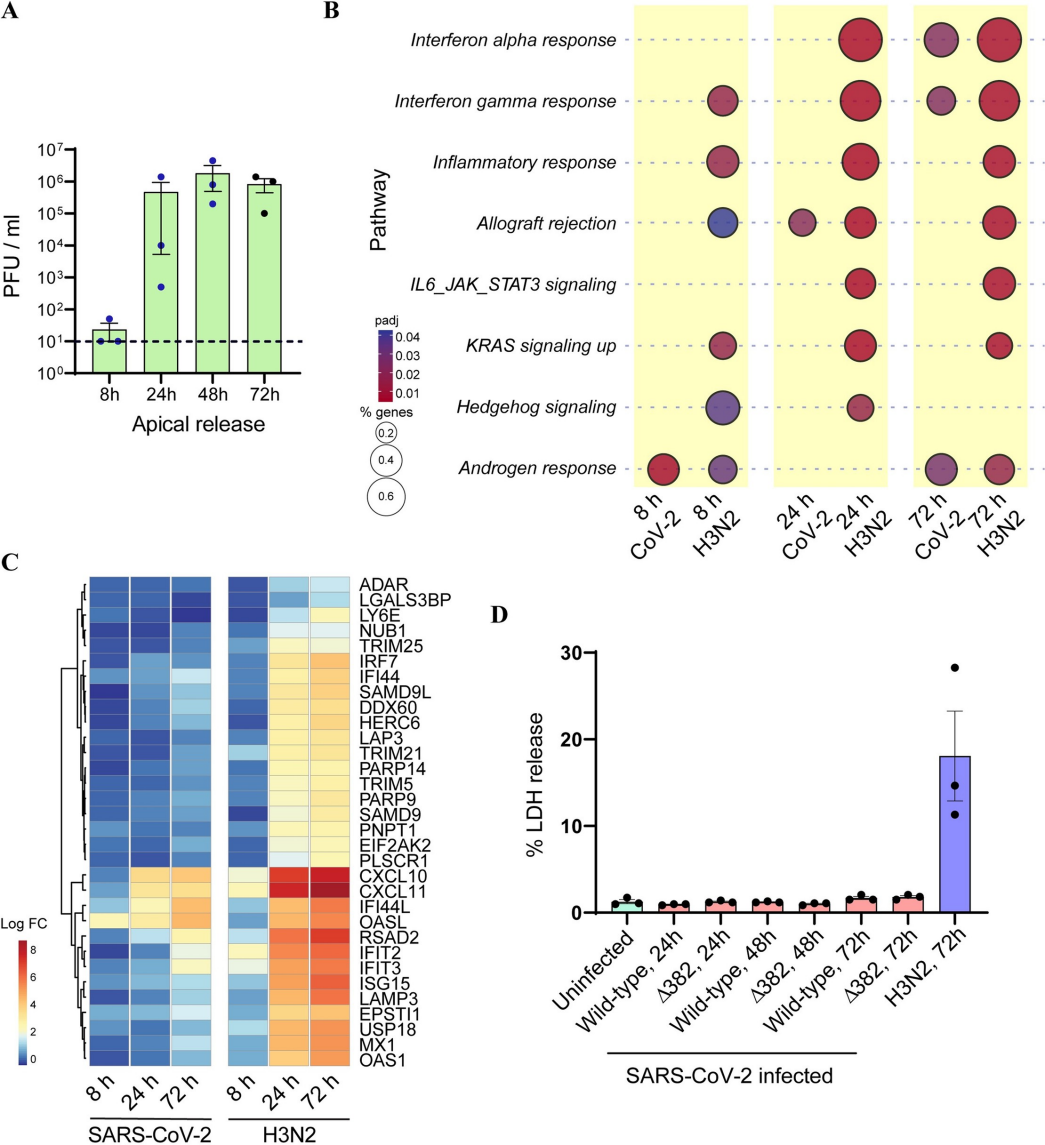
Comparison of transcriptional response to SARS-CoV-2 vs H3N2. (A) H3N2 virus particles released from apical surface of infected NECs, at each of the indicated time-points (n = 3). Each dot represents a different human-donor derived NEC. Data represented as mean ± SEM. Dots in blue indicate data-points which were previously published by Tan KS et al [20]. (B) Bubble-plot of top enriched Hallmark gene-sets upon infection of NECs with H3N2. The corresponding bubble-plots for these same gene-sets upon SARS-CoV-2 infection are included for comparison. The color and size of each bubble is proportional to the adjusted p-value and the percentage of enriched genes from each gene-set, respectively. Only gene-sets with an adjusted p-value <0.05 are shown. (C) Heat-map of key up-regulated DEGs at 72 h after infection of NECs with H3N2, which are specific to Hallmark Inflammatory Response and Interferon Alpha response pathways. The corresponding heat maps for these same genes upon SARS-CoV-2 infection are included for comparison. (D) Percentage of LDH released from apical surface of NECs infected with SARS-CoV-2 wild-type, Δ382 or H3N2 at the indicated time-points. Each dot represents a different human-donor derived NEC. Data represented as mean ± SEM.

As was observed with SARS-CoV-2 infection of NECs, minimal H3N2 release was detected at 8 hpi. Apical viral loads peaked at approximately 2.5 x 10^6^ TCID_50_/ml for SARS-CoV-2 at 72 hpi (Fig 2A) and 1.8 x 10^6^ PFU/ml for H3N2 infected NECs at 48 hpi (Fig 5A), respectively. H3N2 infection was observed to elicit a much stronger and earlier immune response compared to SARS-CoV-2 (Fig 5B and 5C). Genes involved in inflammatory response, IL6-JAK-STAT signaling and KRAS signaling pathways were significantly enriched in H3N2 infected NEC transcriptomes, but was not significantly enriched in transcriptomes from SARS-CoV-2 infected NECs at any time-point (Fig 5B). Notably, genes within the inflammatory response pathway were significantly enriched within 8 hpi of H3N2 infection, highlighting that H3N2 triggers a rapid immune response from NECs (Fig 5B). Further examination of specific genes involved in the inflammatory response and IFN signaling pathways confirmed that H3N2 infection results in the earlier up-regulation of these genes, and to a greater magnitude than observed upon SARS-CoV-2 infection (Fig 5C). H3N2 infection also triggered significant cell death at 72 h, as measured by lactate dehydrogenase (LDH) release into the apical compartment (Fig 5D). In contrast, infection with either wild-type or Δ382 SARS-CoV-2 strains did not result in significant LDH release at any of the post-infection time-points (Fig 5D).

### Δ382 elicits a similar transcriptional response to SARS-CoV-2 upon NEC infection

**Fig 6:**
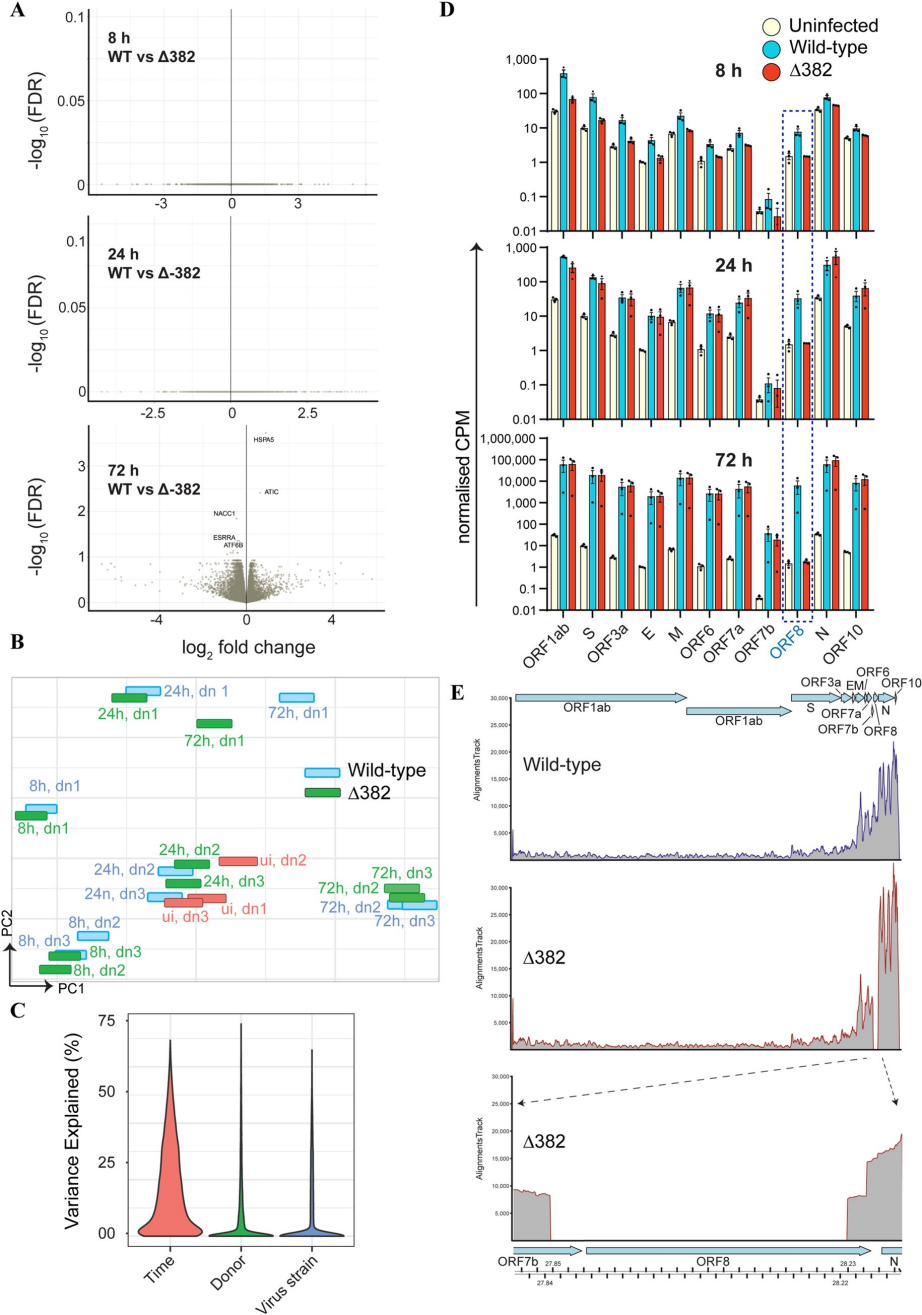
**Similar gene expression profile upon NEC infection with SARS-CoV-2 and Δ382** (A) Volcano plot of DEGs at 8 h, 24 h and 72 h after infection of NECs with wild-type SARS-CoV-2 vs Δ382. Statistically significant DEGs (adjusted p-value < 0.05) are annotated. None of the DEGs have an adjusted p-value < 0.05 and log FC of at least 1.5. (B) PCA plot of transcriptomes from uninfected (ui), wild-type or Δ382 infected donor (dn)-derived NECs, according to gene-expression profiles (C) Percentage of variance explained by post-infection time-point, donor and virus strain for each expressed gene within the transcriptome dataset. (D) Normalized CPM values for SARS-CoV-2 genes detected at each of the indicated time-points from wild-type and Δ382 infected NECs (E) Coverage plot of viral transcripts from wild-type and Δ382 infected NECs at 72 hpi overlaid with the SARS-CoV-2 genome (top inset). Lower panel displays genomic region deleted in Δ382 for which transcript mapping is absent. Each tick represents 10bp on the SARS-CoV-2 genome. All coverage plots are representative images of merged coverage of all three biological replicates, sampled at 1% depth each.

Lastly, we investigated potential differences in the host transcriptional response to infection with wild-type and Δ382. Infection with both strains evoked a similar gene expression profile (Fig 6A). No significant DEGs were observed between NECs infected with the two strains at 8 and 24 hpi. At 72 hpi, only five DEGs had an adjusted p value < 0.05, with all five genes displaying a log_2_ fold change < 1.5 (Fig 6A). Principal component analysis also demonstrated that transcriptomes did not form separate clusters according to strain type (Fig 6B). Rather, it was frequently observed that wild-type and Δ382 infected samples from the same donor and post-infection time-point clustered together (Fig 6B). Virus strain (wild-type vs Δ382) was found to be only a minor contributor to the variation observed across all genes in the transcriptome datasets, with post-infection time-point being the largest contributor (Fig 6C). Sequenced reads from these transcriptome datasets were also mapped to the SARS-CoV-2 genome to quantify expression levels for each of the viral genes. As expected, both wild-type and Δ382 infected samples expressed transcripts corresponding to all SARS-CoV-2 genes with the exception of ORF8 (Fig 6D and 6E).

## Discussion

COVID-19 patients can be asymptomatic or present with a broad spectrum of clinical signs, ranging from mild upper-respiratory tract symptoms to acute-respiratory distress syndrome (ARDS) requiring mechanical ventilation [21,22]. Severe COVID-19 symptoms are linked to an excessive immune response and a pro-inflammatory cytokine storm, which leads to the onset of ARDS and multi-organ system dysfunction [23,24]. Cytokines and chemokines reported to be specifically elevated in the sera of patients with severe disease include IL-6, IL-10, TNFα, MCP-1, MIP-1α and CXCL10 [21,25–27]. Several clinical trials are currently underway for therapeutics which aim to limit this cytokine storm, including the use of IL-6 receptor antagonists [28], α-1 adrenergic receptor inhibitors [29], and the glucocorticoid dexamethasone [30]. Here, we show that airway epithelial cells have a restricted cytokine release profile in response to SARS-CoV-2 infection and are unlikely to be the source of these cytokines elevated in COVID-19 patient serum, with the exception of CXCL10.

This is not due to a general hyporesponsiveness of airway epithelial cells to infection, as previous studies have reported the basolateral release of various inflammatory mediators including IL-6, IL-8 and TNF-α from ALI differentiated airway epithelial cells upon viral infection or toll-like receptor stimulation [31–34]. Rather, the cytokine secretion profile observed upon SARS-CoV-2 infection of NECs is consistent with the attenuated transcriptional response we observed within these cells and reflects a dampened host-response despite productive viral replication. This could be due to active disruption of the host-response by viral proteins, for example via interferon antagonism [35–37]. SARS-CoV-2 may also co-opt specialized membrane structures for virus production [38], sequestering viral RNA and proteins from their detection by cytosolic pattern-recognition receptors. These observations are consistent with the reported lack of interferon induction after *ex vivo* infection of human lung tissue with SARS-CoV-2 [39]. Similarly, another study reported that *in vitro* infection of different cell-types with SARS-CoV-2 resulted in the lower induction of cytokine and chemokine genes as measured by real-time PCR, compared to infection with influenza [40]. Avoiding triggering a strong immune response early during infection could favor virus replication and spread within host tissue. An impaired early response at the primary site of infection may also lead to an eventually prolonged infection and inflammatory response, and thus identifying molecular mechanisms responsible for this dampened response is an important area of further research.

CXCL10 is a chemokine which is sensed by the CXCR3 receptor, expressed on subsets of immune cells including certain monocytes, NK and activated T cells [41,42]. The recruitment of activated T cells and monocytes to the lungs could play an important role in the control of viral infections, as well as in the development of immunopathology via excessive cytokine production and tissue damage. CXCL10 is an interferon-stimulated gene which requires JAK/STAT signaling for its induction. JAK inhibitors have been previously demonstrated to reduce systemic CXCL10 levels in patients with various non-infectious inflammatory diseases [43–45]. It remains to be seen whether the inhibition of CXCL10 production from epithelial cells could have a therapeutic potential in selected COVID-19 patients at increased risk of severe disease, by limiting the infiltration of CXCR3^+^ immune cells into lung tissue. Further work also needs to be done to profile the apical cytokine secretion from infected airway cells, as this could play a role in driving local inflammation on the apical airway side during SARS-CoV-2 infection.

We show that Δ382 does not elicit a significantly different host response from infected cells compared to the wild-type strain, despite the absence of a large genome segment encompassing ORF8. This observation is particularly interesting when juxtaposed with the reduced incidence of severe outcomes and reduced systemic levels of certain cytokines in patients infected with Δ382 [17]. When taken together, this suggests the *in-vivo* differences associated with Δ382 infection are likely an emergent effect from interaction between infected cells and the host immune system, and not a direct consequence of virus strain-specific effects at the cell infection level. In such a scenario, *in-vivo* infection studies using animal models of infection would be needed to further explore the effect of wild-type vs Δ382 strain on virulence.

A previous study reported the inhibition of Type I interferon signaling upon over-expression of ORF8 in HEK293T cells [37]. Our findings instead demonstrated that ORF8 is dispensable for modulating the host transcriptome, including the interferon response. Functional redundancies between multiple SARS-CoV-2 encoded proteins for interferon antagonism could explain this discrepancy [35,36]. On the other hand, our data leaves open the possibility of a post-translational role for ORF8 in modulating the host-response. Affinity purification-mass spectrometry identified the interaction of ORF8 with proteins involved in ER protein quality control and glycosylation pathways [46]. Another study reported the direct interaction between ORF8 and MHC-I upon over-expression in mammalian cells, causing the down-regulation of MHC-I from the cell-surface membrane [47]. ORF8 also contains a predicted immunoglobulin (Ig) domain [48]. Viral encoded Ig-domain containing protein homologs have multiple roles in mediating immune evasion [49].

In conclusion, we establish human donor-derived NECs as a suitable model for further studying host-pathogen interactions upon SARS-CoV-2 infection *in vitro*. Together with recent reports of other *ex vivo* differentiated cell culture systems aiming to recapitulate different aspects of *in vivo* infection conditions [50–55], these model systems would prove critical for evaluating therapeutics and anti-viral agents, exploring the contribution of germ-line encoded donor variation on viral control and host-responses, and assessing the effect of virus mutations on infectivity. This is the first study, to our knowledge, to profile cytokine secretion from SARS-CoV-2 infected epithelial cells, and provides an important basis for dissecting the relative contributions of different cell-types in lung airway tissue to the immunopathology observed in COVID-19 patients.

## Materials and methods

### Ethics statement

Approval to conduct this study was obtained from the National Healthcare Group Domain-Specific Board of Singapore (DSRB Ref: D/11/228) and institutional review board of the National University of Singapore (IRB Ref: NUS-IRB-2020-33). Written consent was obtained from donors prior to the collection of the tissue biopsies.

### Derivation of human nasal epithelial stem/progenitor cells (hNESPCs) and *in-vitro* differentiation of hNECs

Nasal biopsies were obtained from the inferior turbinate of healthy donors undergoing septal deviation surgery. At the time of collection, all subjects were free of symptoms of upper respiratory tract infection (URTI), and did not take any forms of glucocorticoids (GC) or antibiotics within three months before the study. The hNESPCs were isolated and enriched from the tissue biopsies according to a previously standardized protocol [18,56]. Briefly, primary cells were subjected to isolation for selection of hNESPCs, which were enriched and expanded with Dulbecco's Modified Eagle Medium: Nutrient Mixture F-12 (DMEM/F12) (Gibco-Invitrogen) containing 10 ng/mL of human epithelial growth factor (EGF, Gibco-Invitrogen), 5 μg/mL of insulin (Sigma), 0.1 nM of cholera toxin (Sigma), 0.5 μg/mL of hydrocortisone (Sigma), 2 nM/ml of 3, 3’, 5- triiodo- L- thyronine (T3) (Sigma), 10 μL/mL of N-2 supplement (Gibco-Invitrogen) and 100 IU/ml of antibiotic-antimycotic (Gibco-Invitrogen). The expanded hNESPCs were then transferred onto 12-well 0.4 μm transwell inserts (Corning). Once confluent, growth medium from both apical and basal chamber was discarded and 700 μl of PneumaCult™-ALI Medium with inducer supplements (STEMCELL Technologies Inc.) was added to the basal chamber to establish ALI conditions. The cells were cultured in ALI culture for 4 weeks, with media change every 2–3 days. Fully differentiated hNECs after 3–4 weeks of differentiation were used for the SARS-CoV-2 infection.

### Tissue specimen processing

All nasal biopsy specimens were processed as previously reported [57]. Briefly specimens were embedded in paraffin and sectioned at 4 μm with a Leica microtome (Leica). Paraffin sections of nasal samples were dewaxed with xylene and ethanol, antigen retrieved with boiling sodium citrate buffer (pH 6) and subjected to immunofluorescence (IF) staining.

### Cross-section of transwell hNECs preparation

Cross-section of hNECs on transwell were paraffin embedded for processing and subjected to IF staining. Briefly, hNECs on transwell were washed twice with 1x PBS. Cells were then fixed with 4% PFA for 10 min at room temperature. The transwell membrane was washed with 1x PBS, removed from the inserts and placed into cassettes for subsequent dehydration in ethanol. The membrane was then treated with 2x xylene for 10 min and 2x with liquid paraffin for 30 min each. The solidified membranes were embedded in a paraffin boat taking into account orientation for sectioning. 4–5 μM sections were cut according to the standard procedures, mounted on glass slides, and allowed to dry for subsequent processing.

### Immunofluorescence (IF) staining

Rabbit polyclonal antibodies against ACE2 [21115-1-AP] (Proteintech), rabbit polyclonal antibody against TMPRSS2 [14437-1-AP] (Proteintech) and mouse monoclonal antibody against βIV-Tubulin [ab11315] (Abcam) were used at 1:500, 1:100, and 1:800, respectively. Paraffin embedded sections were dewaxed and antigen retrieved prior to IF staining. All sections were treated with 0.1% TritonX-100 for 10 min at room temperature, followed by three 1x PBS washes. Sections were blocked with 10% goat serum for 30 min at room temperature and incubated with a primary antibody solution (diluted with 1% goat serum) overnight at 4°C in the dark. Sections were then incubated for 1 h with Alexa Fluor 488- or Alexa Fluor 594- conjugated secondary antibodies in the dark at room temperature. Upon discarding the stain, coverslips were mounted on slides by using SlowFade Gold antifade reagent with 4’ 6-diamidino-2-phenylindole (DAPI) (Life Technologies). The slides were analyzed with a fluorescent microscope (Olympus IX51).

To validate specificity of the ACE2 and TMRPSS2 polyclonal antibodies, HEK293T cells were transfected with pFUGW-EF1a-ACE2 using FuGENE transfection reagent (Promega), and VeroE6 cells were transfected with pCAGGS-TMPRSS2 using Lipofectamine3000 transfection reagent (ThermoFisher). After 24 h of transfection, cells were fixed in 4% PFA, permeabilized with 0.1% TritonX-100 and IF staining carried out as described above.

### Virus culture, infection and quantification

SARS-CoV-2 and Δ382 were isolated from COVID-19 patients in Singapore, as reported previously [14]. NECs differentiated on 12-well transwell plates were washed with dPBS, and infected from the apical surface with 100 μl of viral inoculum for 1 h, the apical surface was rinsed twice in dPBS and then incubated in a tissue culture incubator at 37°C. At each time-point, 100 μl of dPBS was added to the apical side, incubated for 10 min, and then aspirated and stored for virus titration. Basolateral medium was directly harvested for cytokine analysis and virus titration. For virus titration, supernatant was overlaid on Vero-E6 cells and the 50% tissue culture infective dose was calculated after incubation for 4 days. RNA was extracted from cell lysates using an EZNA RNA extraction kit (Omega BioTek) according to manufacturer’s instructions. Viral copy numbers were first assayed using a real-time reverse-transcription PCR reaction with primers targeting the RdRp gene, and then quantified using a standard curve as previously described [58].

### Cytokine quantification

Multiplexed cytokine assays were performed using a Bio-Plex Pro Human cytokine screening panel (Biorad) containing 48 human cytokines. Samples were diluted 4x prior to assay, except for GRO-α quantification which was performed on 10x diluted samples. Spectral intensities were quantified on a MagPix machine (Luminex Corporation). Cytokine concentrations were calculated by interpolating from a standard curve via 5PL curve fitting. Samples below the detection limit were assigned a value of 1 to enable the use of log scale for calculating fold changes and graphing. IL-11 was quantified using a Human IL-11 Quantikine ELISA Kit according to manufacturer’s instructions (R&D Systems). Statistical analysis was performed on log_10_ transformed values using two-way ANOVA test with Tukey correction. To identify cytokines secreted by NECs in the uninfected state, the following criteria were used: > 10 fold increase in cytokine signal at 72 h compared to media only control, and adjusted p-value < 0.01 between cytokine signal at 72 h compared to media only control, obtained via two-way ANOVA test with Tukey correction. To identify cytokines significantly increased in concentration upon infection, adjusted p-value < 0.01 between cytokine signal at 72 h compared to uninfected control at 72 h was used as the threshold criteria, obtained via two-way ANOVA test with Tukey correction.

### Quantification of cell death

Lactate Dehydrogenase (LDH) released into the apical compartment was measured as an indicator of cell death at different time-points post-infection, and expressed as a percentage of total LDH released upon lysis of the NECs with 0.1% Triton in PBS. Quantification was performed with an LDH Cytotoxicity Detection Kit (Takara Bio) according to manufacturer’s instructions.

### Library preparation and Next Generation Sequencing (NGS)

Total RNA was extracted as described above, and quantified on an Agilent 2100 Bioanalyzer. All samples had a RIN score > 7. Library construction was performed with 1 μg total RNA using a TruSeq Stranded Total Human RNA Prep kit (Illumina) according to manufacturer’s instructions, and included cytoplasmic rRNA depletion. A total of 21 cDNA samples were pooled, and sequenced on three lanes using a HiSeq4000 platform (Illumina).

### Sequences and data

The sequence and coordinates for SARS-CoV2 (NC_045512.2) and the human genome (GRCh38.p12) were obtained from GenBank. For the human genome, pseudogenes, rRNA and mitochondrial genes were filtered out from the gtf file. RNASeq data for H3N2 were obtained from a previously study, in raw fastq form [20]. Sequence references for wild-type and Δ382 strain used in this study were accessed from the GISAID database under accession codes EPI_ISL_407987 and EPI_ISL_414378, respectively.

### Read mapping and QC

All sequences were examined by FastQC (v0.11.9) and MultiQC (v.1.0.dev0), passing QC with Phred Score > 30. The human genome was indexed with default parameters in STAR (v2.7.5a), and the SARS-CoV-2 genome was indexed with *genomeSAindexNbases* = 6, according to the size of its genome. The same paired-end sequence data were then mapped separately with STAR to the viral and human genomes, with default parameters. Alignment quality was assessed with QualiMap (v2.2.2a) and MultiQC, and count tables were generated with *FeatureCounts* within the R package *Rsubread* (v2.0.1), under R (v.3.6.1). Filtering of genes were first based on donor bias: edgeR’s ANOVA-like test (edgeR Manual 3.2.6) (edgeR v3.28) was used to compute genes that were strongly biased (cut-off: FDR < 0.05 & | 2 x logFC | > 10; each logFC coming from one donor). Then the function *filterByExpr* was used to remove lowly expressed genes, with default parameters and grouping by a combined time/virus-used variables (SARS-CoV-2) or time (H3N2).

### DEG, PC and gene-set enrichment analysis

Differential Gene Expression Analysis was performed using edgeR’s *glmQLFTest*, with a model matrix of (~Donor + Group), where Group is defined as VirusUsed + Time. Pairwise comparisons were made with each time-point vs control (both SARS-CoV2 and H3N2), as well as wild-type vs Δ382. Paired comparisons were performed taking into account effects of Donor (edgeR Manual 3.4.2). For host genes, only coding sequences were used in the comparisons. For viral genes, the analysis was performed using host and viral genes to get a more accurate representation of the library size. For gene expression analysis at the individual level, after filtering for low expression and skewed donor effect (as mentioned above), the raw counts were used to calculate the fold change; a pseudocount of 1 added to all raw counts, and the log2 fold-change was calculated for the ratio (infected/uninfected). This data was then used to generate the individual level heatmaps.

Principle Components were calculated with host genes only, using *prcomp* in the base R stats package. In order to quantify the contributions of various co-variables (Time, Virus Used and Donor) to the variance, *fitExtractVarPartModel* function in the *variancePartition* package (v1.16.1) was used to calculate the percentage contribution of each co-variable to each gene.

Gene-set enrichment analysis was performed using the package *fgsea* (v1.12.0). Gene-sets were obtained from package *msigdbr* (v7.1.1) and the hallmark pathways were used for both H3N2 and SARS-CoV2 datasets. Ranked lists were computed using the sign of the logFC x FDR. Percentage of genes contributing to the pathways utilizes the number of genes computed as *leadingEdge* genes, in comparison to the total number of genes in that gene-set available in our dataset. For plotting pathways enriched upon SARS-CoV-2 infection (Fig 4B) and H3N2 infection (Fig 5B), the top 5 significant pathways (adjusted p-value <0.05) sorted by positive normalized enrichment score (NES) at 8 h and 24 h time-points were used for the bubble-plot generation. Graphics were created using GraphPad Prism 8, ggplot2 (v3.2.1), and pheatmap (v1.0.12).

### Coverage plots

Output SAM files from STAR Alignment (with tag—outSAMunmapped Within) were further filtered to contain only unmapped reads, and converted back to .fastq files using scripts provided in *Virdetect [59]*. .fastq files were then re-mapped to the SARS-CoV2 genome, and bam files were sampled at 1% depth using samtools. Sampled coverage data were visualized using the *Gviz* package (1.30.3). The consensus SARS-CoV-2 sequence from infected NEC RNA sequencing data was generated by taking bam files of all viral reads (filtered as non-human reads and subsequently aligned to Wuhan-Hu-1 (NC_045512.2) reference) using the mpileup function from samtools, and calling variants using bcftools. The consensus sequence was then generated using the function consensus in bcftools.

## Supporting information

S1 Fig Specificity of anti-ACE2 and anti-TMPRSS2 antibodies used for immunofluorescence assay. Secondary antibody only staining controls for (A) upper airway tissue section, and (B) hNEC cross-section. (C) Immunofluorescence staining for expression of TMPRSS2 (red) in VeroE6 untransfected cells, or VeroE6 transfected with plasmid over-expressing TMPRSS2, as indicated below each panel. (D) Immunofluorescence staining for expression of ACE2 (red) in HEK293T untransfected cells, or HEK293T transfected with plasmid over-expressing ACE2, as indicated below each panel. Nuclear staining with DAPI is shown in blue.
(TIF)

S2 Fig Donor specific transcriptional response to SARS-CoV-2 infection from NECs. (A) Heat-map of top up-regulated interferon-related genes after infection of NECs with wild-type SARS-CoV-2, displayed for each individual donor derived NEC. (B) Virus particles released from the apical surface of infected NECs, labelled with the corresponding donor code (C) Negative correlation between average IFN-related gene induction at 8 hpi and apical viral yield at 72 hpi.
(TIF)

S1 Table Nucleotide sequence and amino acid differences in wild-type and Δ382 strains used in this study. (DOCX)
