# Supplementary figures and images for: Infection of human Nasal Epithelial Cells with SARS-CoV-2 and a 382-nt deletion isolate lacking ORF8 reveals similar viral kinetics and host transcriptional profiles
Source: PLoS Pathog. 2020 Dec 7;16(12):e1009130. doi: 10.1371/journal.ppat.1009130 (PMC7746279; doi:10.1371/journal.ppat.1009130)

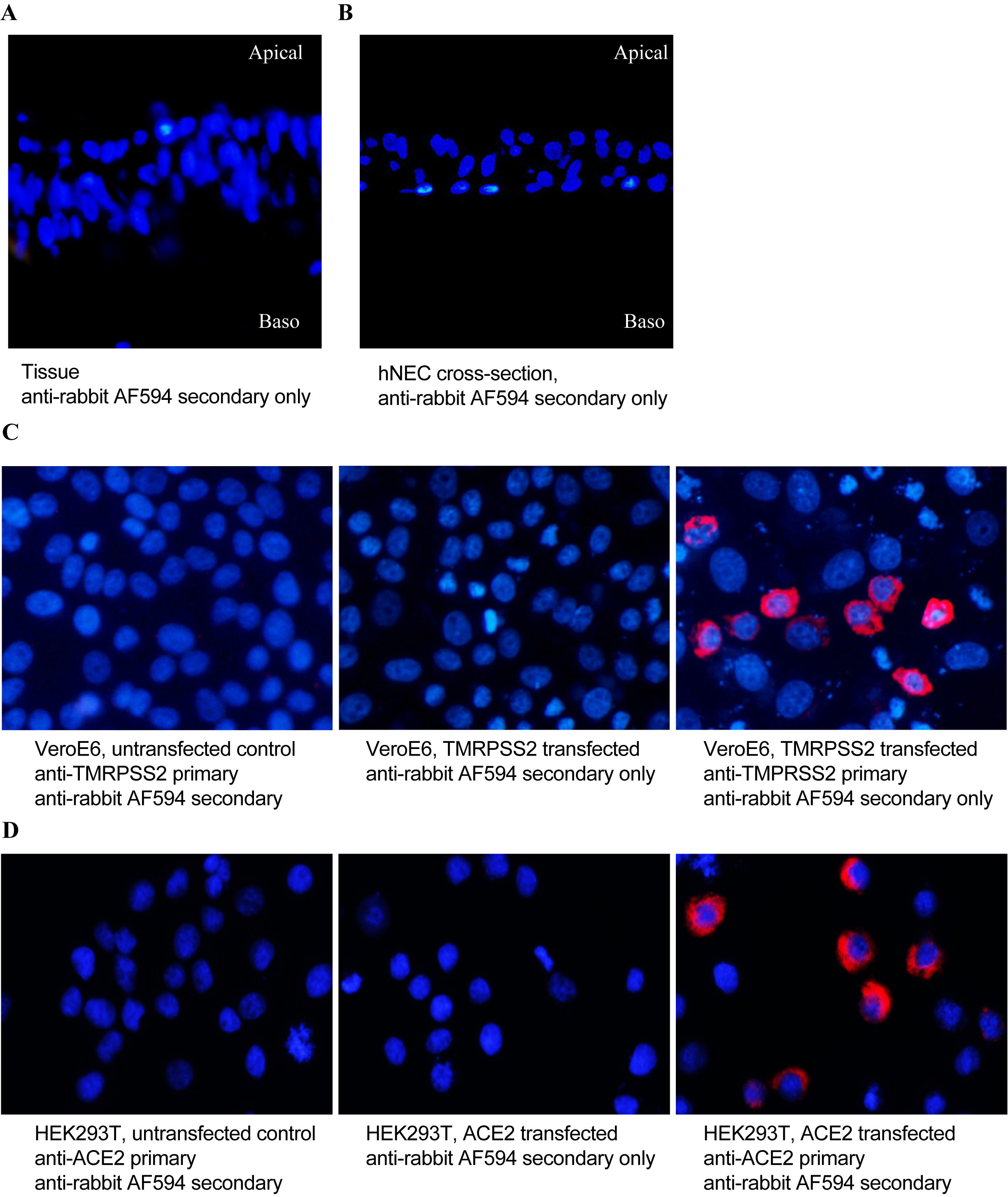

Supplement: S1 Fig — Secondary antibody only staining controls for (A) upper airway tissue section, and (B) hNEC cross-section. (C) Immunofluorescence staining for expression of TMPRSS2 (red) in VeroE6 untransfected cells, or VeroE6 transfected with plasmid over-expressing TMPRSS2, as indicated below each panel. (D) Immunofluorescence staining for expression of ACE2 (red) in HEK293T untransfected cells, or HEK293T transfected with plasmid over-expressing ACE2, as indicated below each panel. Nuclear staining with DAPI is shown in blue. (TIF) [file ppat.1009130.s001.tif]

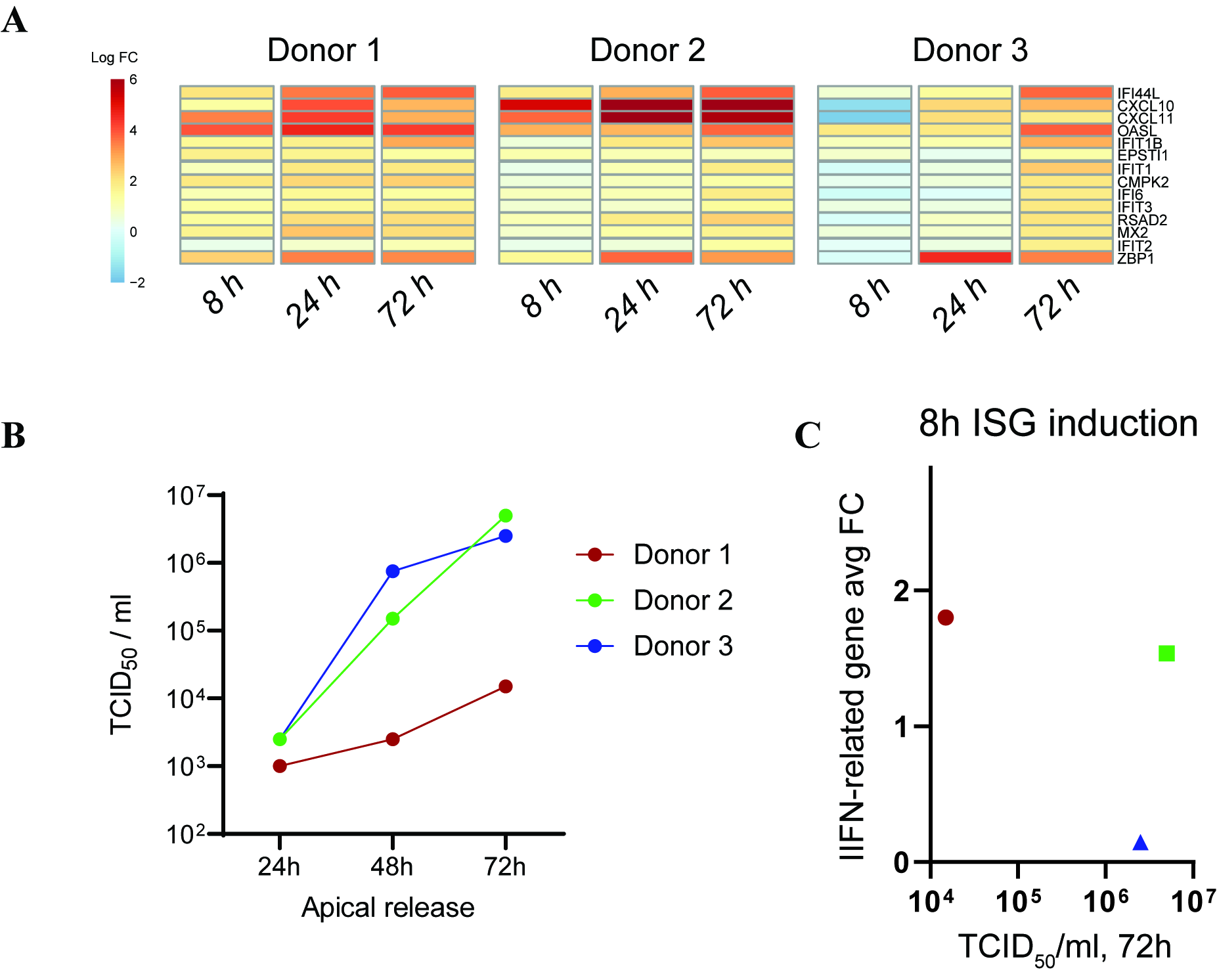

Supplement: S2 Fig — (A) Heat-map of top up-regulated interferon-related genes after infection of NECs with wild-type SARS-CoV-2, displayed for each individual donor derived NEC. (B) Virus particles released from the apical surface of infected NECs, labelled with the corresponding donor code (C) Negative correlation between average IFN-related gene induction at 8 hpi and apical viral yield at 72 hpi. (TIF) [file ppat.1009130.s002.tif]
