# Supplementary material for: Infection of human Nasal Epithelial Cells with SARS-CoV-2 and a 382-nt deletion isolate lacking ORF8 reveals similar viral kinetics and host transcriptional profiles
Source: PLoS Pathog. 2020 Dec 7;16(12):e1009130. doi: 10.1371/journal.ppat.1009130 (PMC7746279; doi:10.1371/journal.ppat.1009130)
Supplement: S1 Table — (DOCX) [file ppat.1009130.s003.docx]

**S1 Table: Nucleotide sequence and amino acid differences in wild-type and Δ382 strains used in this study.**

| Position | Gene | GISAID ref | | Tr consensus | | AA change |
| --- | --- | --- | --- | --- | --- | --- |
|  |  | **Wild-type** | **Δ382** | **Wild-type** | **Δ382** |  |
| 8,782 | ORF1AB | . | T | . | T | Silent |
| 23,525 | S | . | . | T | T | H to Y |
| 24,011 | S | . | C | . | C | F to L in Δ382 |
| 25, 381 | S | . | . | . | C | Silent |
| 25,568 | ORF3A | . | A | . | A | A to D in Δ382 |
| 27,147 | M | C | . | C | . | D to H in wild-type |
| 27,848-28,229 | ORF7A, ORF8 | . | Absent | . | Absent |  |

GISAID ref: sequence derived from GISAID reference, Tr consensus: sequence derived from NEC transcriptomes generated in this study. Fields highlighted in yellow represent amino acid differences between wild-type and Δ382 strain.
